# Viewing distance and character size in the use of smartphones across the lifespan

**DOI:** 10.1371/journal.pone.0282947

**Published:** 2023-04-12

**Authors:** Laura Boccardo, Massimo Gurioli, Paolo Antonino Grasso

**Affiliations:** 1 Department of Physics and Astronomy, School of Optics and Optometry, University of Florence, Sesto Fiorentino (FI), Italy; 2 Institute for Research and Studies in Optics and Optometry (IRSOO), Vinci (FI), Italy; University of New South Wales, AUSTRALIA

## Abstract

The use of smartphones has seen an extraordinary growth in recent years, thus the understanding of visual habits associated with the use of such devices across the lifespan is becoming important. In the present study we measured viewing distance and character size in a group of non-presbyopes (n = 157) and a group of presbyopes (n = 60) while participants read a simple text message on their smartphone. Results showed that non-presbyopes use shorter viewing distances as compared to presbyopes, a behavior causing a significantly higher accommodative demand. Presbyopes also use larger character sizes and this behavior is more evident whenever difficulties in near vision emerge in the Near Activity Visual Questionnaire (NAVQ, Italian version). Nevertheless, the two groups did not differ in the measurement of angular size subtended by the smallest detail of the letters. Overall, our data reveal that non-presbyopes and presbyopes have different visual habits when using a smartphone. These differences should be considered when determining the best near correction.

## Introduction

During the last decade, the use of smartphones and tablets has seen a tremendous increase, moving from being a prerogative of young people to a diffuse daily practice across the entire lifespan. Almost 75% of adults in Italy spend more than 3 hours a day using their smartphone, with 14% spending more than 8 hours a day [1]. Further, it is estimated that, on average, users check their smartphones more than 200 times per day which means every 4 minutes assuming a 16-hour day. No doubts that the use of smartphones is becoming more and more ubiquitous in daily living [2].

Smartphones can be of a great help in the optimization of our time as the large-scale introduction of such devices has increased and eased communications in everyday lives. However, their worldwide spread has dramatically changed our visual habits and attention should be paid to the effects in ocular health. For instance, the relatively small screen of these devices may lead users to reduce usual working distance, as shown in previous studies reporting closer distances as compared to the traditional point used for printed materials [3, 4]. It is expected that this behavior may vary with age due to the reduced accommodative power in older individuals.

The present study aimed at clarifying these points to shed light on visual habits associated with the use of smartphones across different age groups. In particular, we selected a group of non-presbyopes (age < 40) and a group of presbyopes (age > 40). Presbyopia is a refractive error condition characterized by a reduced accommodative power of the lens which makes it hard for middle-aged and older adults to focus on nearby objects [5]. Visual habits were defined as the working distance and character size used while reading a text message on their personal smartphone. The two measurements allowed us to determine the corresponding angular size in the two groups. First, we expected to confirm a difference in the measurement of reading distance adopted by non-presbyopes and presbyopes during smartphones usage [4]. Secondly, we aimed to investigate whether differences in reading distance are also accompanied by differences in character dimension adopted by the two groups.

## Materials and methods

### Participants

To calculate the sample size we performed a power analysis using G*Power 3 Software [6], which indicated that a sample of 214 participants would be needed to detect medium effects (d = 0.5) with 95% power, an alpha level of 0.05 and an allocation ratio of 2.5 between the two groups of participants (i.e., non-presbyopes and presbyopes). The allocation ratio was here estimated to be different than 1 as the study mainly employed students recruited at the University of Florence and thus way more likely to be below 40 years old. Participants above 40 years old were mainly parents and relatives of students involved in data collection.

A total of 217 participants took part in the study. Inclusion criteria consisted in the absence of self-reported ocular pathologies. All participants provided written informed consent for their participation to the study. The research was approved by the local ethics committee (“*Commissione per l’Etica della Ricerca”*, University of Florence, 23^rd^ December 2020, n. 131) and was conducted in accordance with the Declaration of Helsinki.

### Procedure and data collection

**Fig 1:**
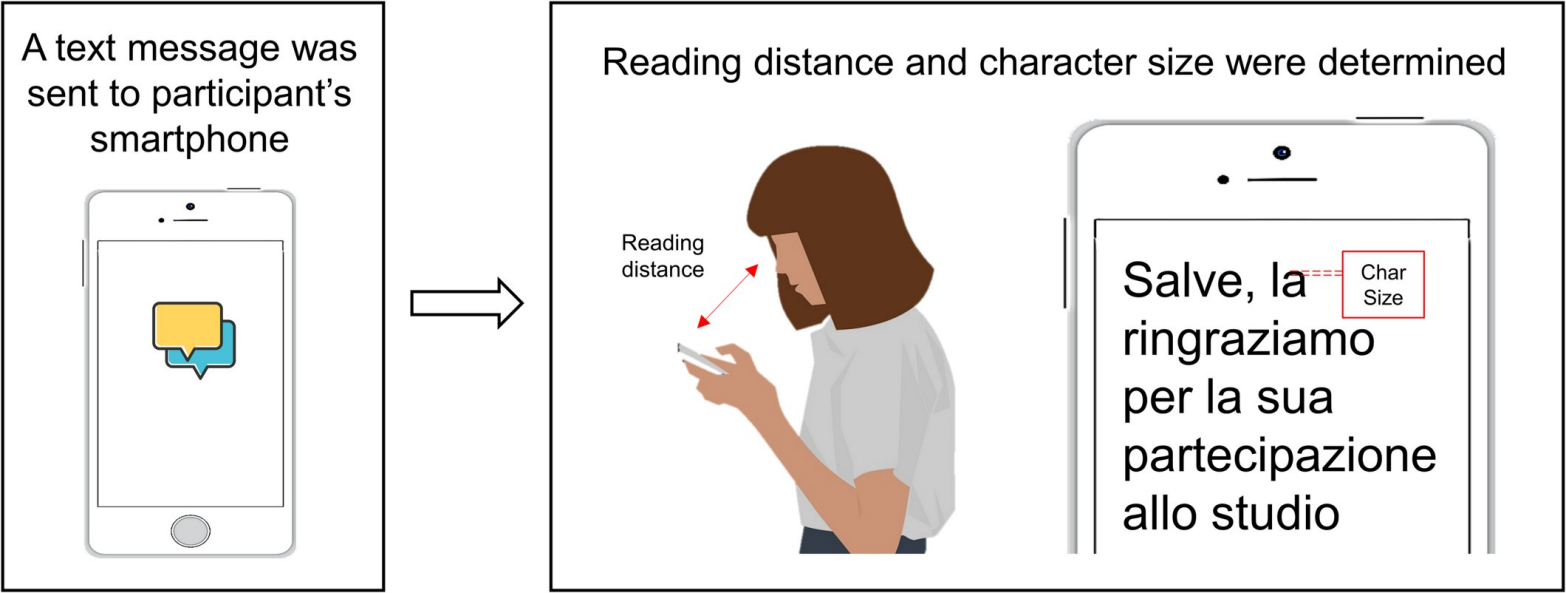
Experimental procedure. Participants received a text message and were instructed to read it. Reading distance and character size were obtained.

Data collection consisted in participants wearing their habitual refractive correction (spectacles or contact lenses) while reading a typical text message on their own smartphone. More specifically, participants received on their personal smartphone a text message written in Italian and were instructed to read it while keeping the device at the usual reading distance (Fig 1).

The distance between the smartphone and the corneal vertex was determined using a tape measure with an approximation to the nearest millimeter. In a subgroup of participants (i.e., 32 participants), we calculated the reading distance both using tape measure and through a specific smartphone application (Myopia app; Innovattic) which, after a quick and easy calibration, estimated the distance of use through the integrated front camera. Participants kept the application active in background for 24 hours and provided an average usage distance along the time of recording. We used this alternative method with the sole scope of benchmarking measurements obtained with tape measure, to which refers all the other measurements reported hereafter.

We then derived the accommodative demand related to individual reading distance, as follows:

$$K=\frac{1}{d}$$

where *K* is the vergence in diopters (D) and *d* is the viewing distance in meters.

Character dimension adopted by each participants was determined by evaluating the number of pixels composing the height of lower-case letters of the text message that did not contain ascenders and descenders (e.g., m, o, e). We transformed the obtained pixels’ values in millimeters using the following formula:

$$h=h_{pxl}\times \frac{diag_{mm}}{diag_{pxl}}$$

where *h*_*pxl*_ is the character dimension in pixel while *diag*_*mm*_ and *diag*_*pxl*_ are measures of the diagonal of the screen in millimeters and pixels which were obtained from smartphones’ data sheet.

The combination of reading distance and character dimension allowed us to evaluate the subtended natural angle of resolution (NAR), which was calculated as follow:

$$NAR=\tan^{-1}\left(\frac{h}{5d}\right)\times 60$$

where *h* is the character dimension and *d* is the reading distance with both measurements reported in millimeters and with distance multiplied by a factor of five to obtain the NAR corresponding to the smallest stimulus detail. The logarithm of the inverse NAR (i.e., LogNAR) was implemented in statistical analyses [7].

To measure near visual abilities, presbyopes also completed the Italian version of the Near Activity Visual Questionnaire (NAVQ) which allowed an assessment of individual difficulties in near vision [8, 9]. According to the criterion of Buckhurst and colleagues [8], a total score of 10 or greater (range: 0–30) has to be considered as reflecting near vision difficulties.

### Statistical analysis

We performed statistical analyses using ANOVAs or two-tailed independent sample t-tests (using JASP, Version 0.14.1.0). When assumptions of normality or equal variance across samples were not met, the corresponding non-parametric tests were employed.

## Results

**Fig 2:**
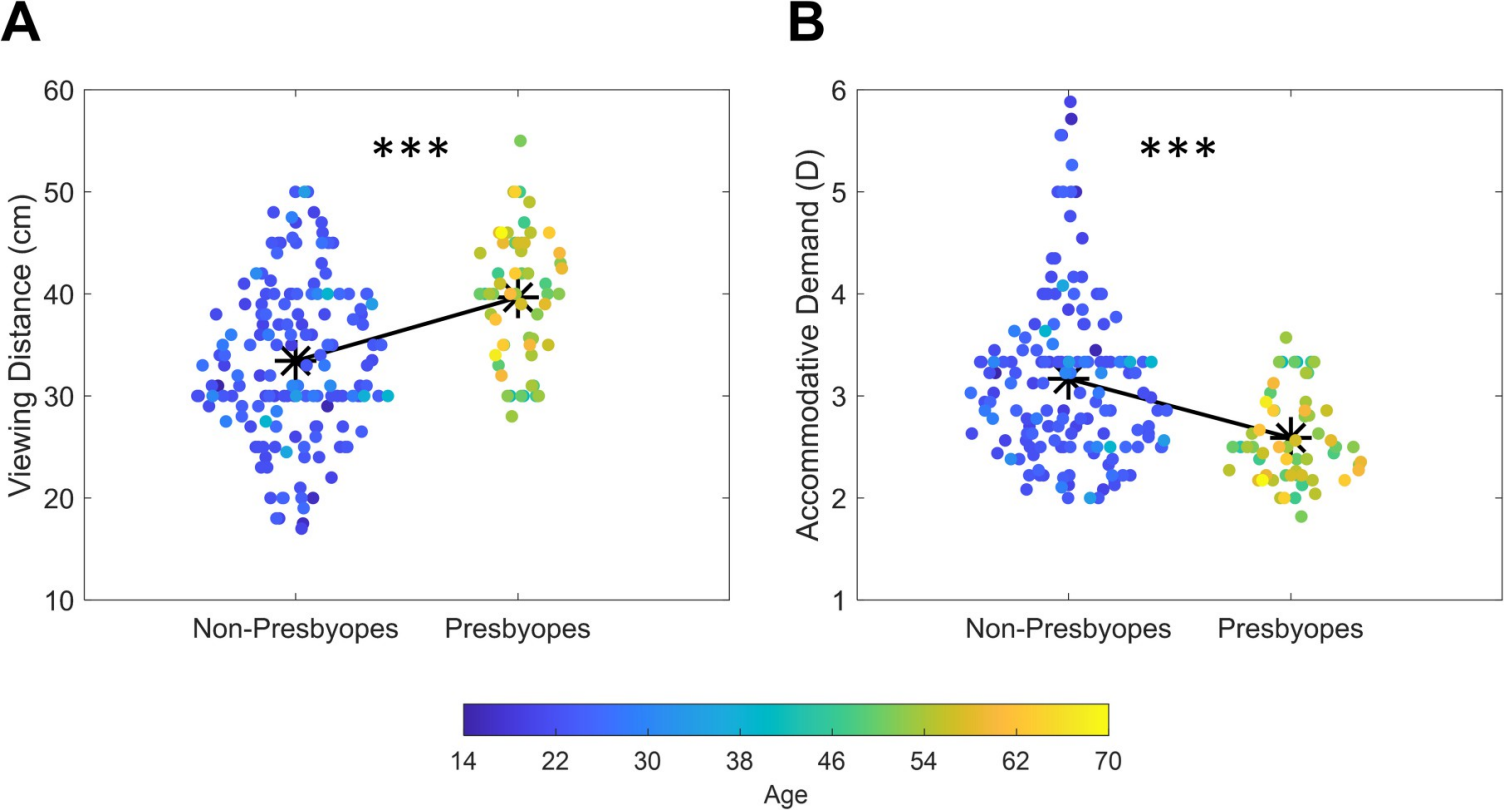
Scatterplot depicting values of reading distance (A) and accommodative demand (B) for non-presbyopes and presbyopes. Asterisks depicts average values while different colors represent different ages (*** = p < 0.001).

As a first procedure, we split the sample into two sub-groups composed of non-presbyopes (i.e., age below 40 years; 157 participants; mean age: 23.7 years; std: 4.13 years; range: 14–39) and presbyopes (i.e., age above 40 years; 60 participants; mean age: 53.9 years; std: 5.98 years; range: 41–70). Then, we compared values of reading distance between the two groups. Results revealed a higher reading distance for presbyopes (mean = 39.7 cm; std = 6.3 cm) than non-presbyopes (mean = 33.4 cm; std = 7.6 cm; Fig 2A). A t-test indicated that this difference was statistically significant (*t*_(215)_ = -5.59; *p* < 0.001; d = -0.85). Consequently, accommodative demand was found to be higher for non-presbyopes (3.17 D) than presbyopes (2.59 D; Fig 2B). Given previous reports of an association between reading distance and forearm length (i.e., Harmon distance) we tested if this held true also in our sample. We then compared reading distance measurements between males and females assuming males having, on average, longer forearm lengths leading to adopt higher reading distance. As expected, results showed that males used, on average, a higher reading distance as compared to females (males: 38.1 cm, std = 7.1 cm, females: 32.8 cm, std = 7.6 cm; *t*_(215)_ = 5.22; *p* < 0.001; d = 0.71). We then conducted a 2 x 2 ANOVA with factors Sex (Males and Females) and Group (Non-Presbyopes and Presbyopes) to check for any specificity of the effect of sex in the two groups of participants. The analysis revealed a significant main effect of Sex (*F*_(1, 213)_ = 22.382; *p* < 0.001; *ƞ*^*2*^ = 0.08) and Group (*F*_(1, 213)_ = 36.075; *p* < 0.001; *ƞ*^*2*^ = 0.13) but no interaction between the two (*F*_(1, 213)_ = 1.30; *p* = 0.255) suggesting that the difference in reading distance between males and females was evident both in non-presbyopes and presbyopes.

**Fig 3:**
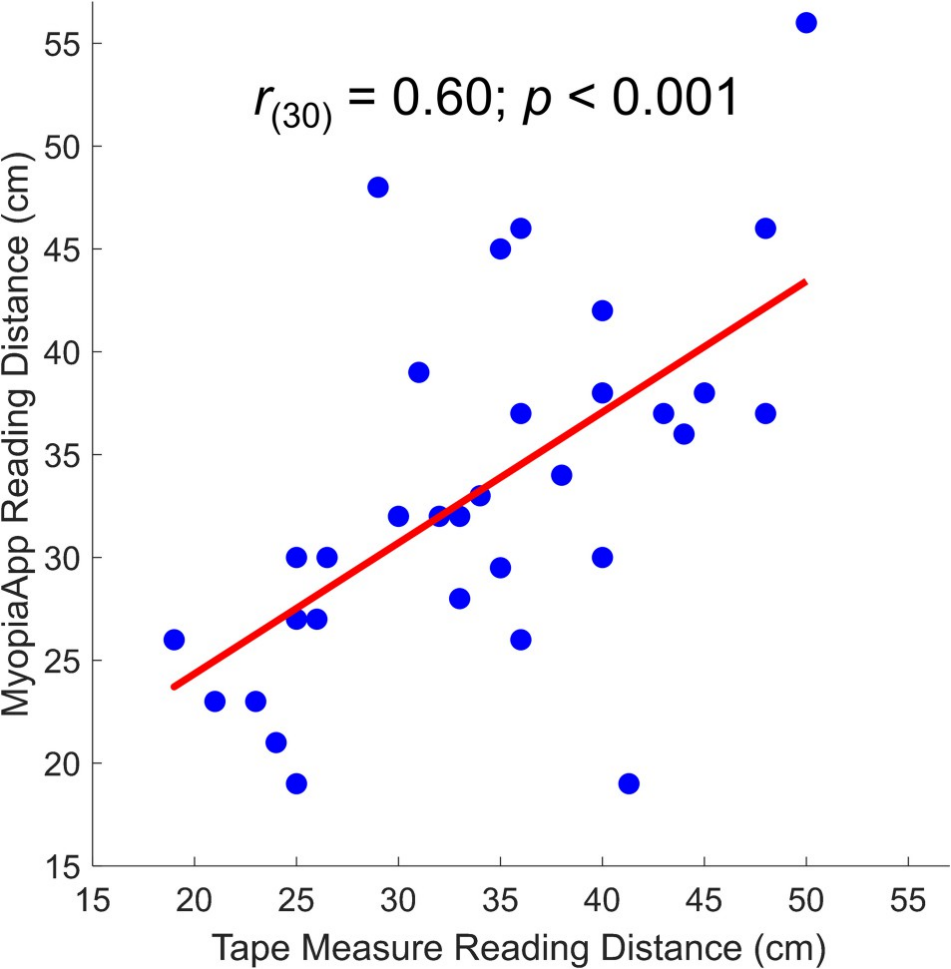
Correlations between reading distances obtained using tape measure and the use of a dedicated smartphone application.

To test the reliability of tape measure we compared reading distances obtained using tape measure with reading distances estimated through a dedicated smartphone application (see *Procedure and Data Collection* section above for further details) in a subgroup of participants. Results showed that the two measurements were highly correlated (Pearson; *r*_(30)_ = 0.60; *p* < 0.001; Fig 3).

**Fig 4:**
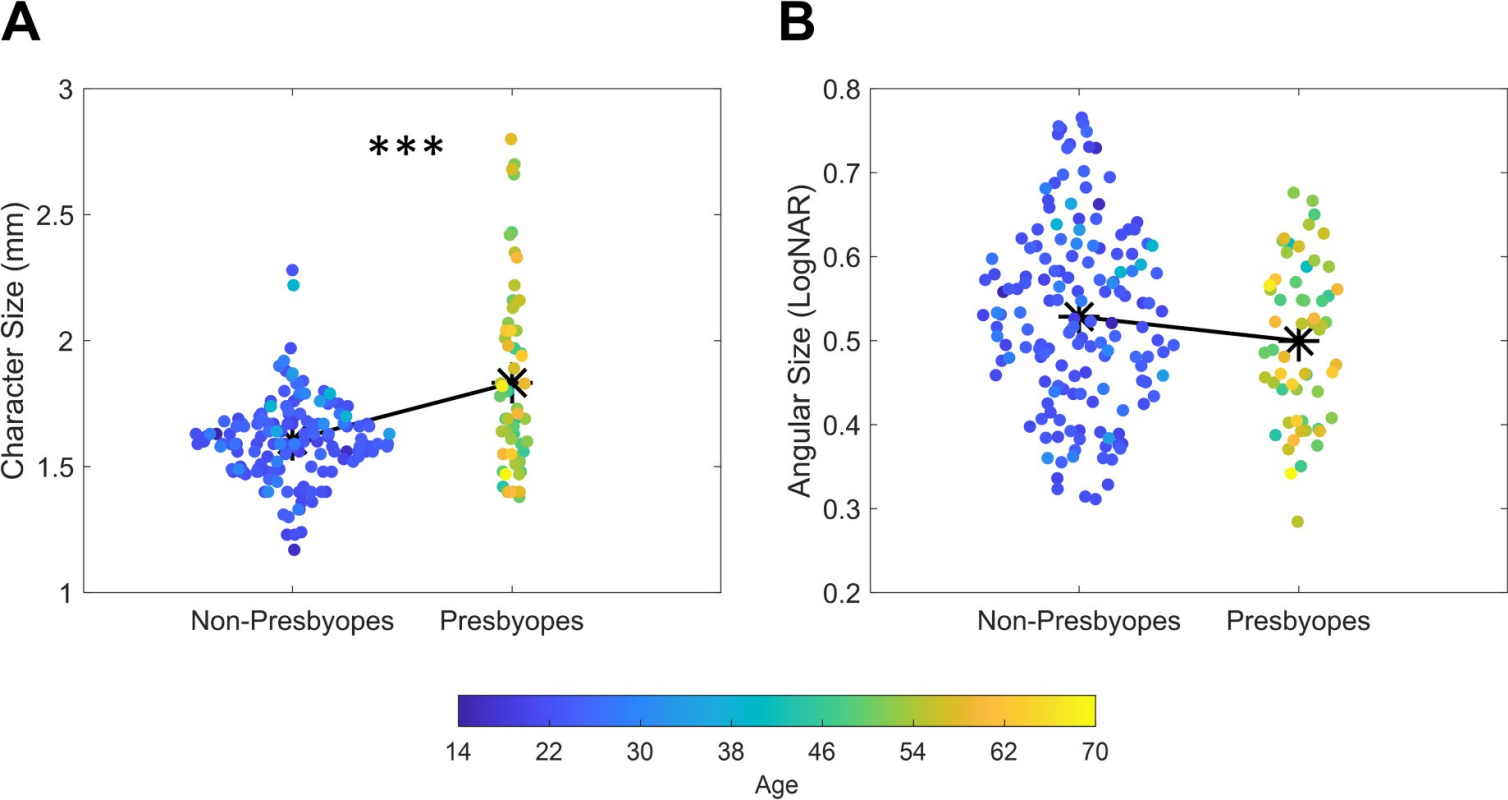
Scatterplot depicting values of character size (A) and angular size (B) for non-presbyopes and presbyopes. Asterisks depicts average values while different colors represent different ages (*** = p < 0.001).

We also compared character sizes across the group of non-presbyopes and presbyopes. Results revealed that non-presbyopes used smaller character sizes (*Md* = 1.59 mm; IQR = 0.19) as compared to presbyopes (*Md* = 1.72 mm; IQR = 0.49) with this difference being significant at the Mann-Whitney test (*U* = 3121; *p* < 0.001; *r* = -0.337; Fig 4A). In this case, no difference between males and females was instead evident (*t*_(215)_ = 1.19; *p =* 0.235).

Finally, also reading angular size showed no difference between the two groups (*t*_(215)_ = 1.83; *p* = 0.07; Fig 4B) with non-presbyopes having very similar measurements (0.53 LogNAR) to those reported by presbyopes (0.5 LogNAR).

We also checked for any difference between presbyopes wearing no refractive correction, single vision lenses, progressive glasses or contact lenses. Results revealed no significant difference between the four groups in angular size (*F*_(2, 57)_ = 0.845; *p =* 0.435), reading distance (*F*_(2, 57)_ = 0.057; *p =* 0.945) and character size (*F*_(2, 57)_ = 0.473; *p =* 0.626) suggesting that different types of correction did not produce significant changes in visual habits.

We also investigate differences in screen dimensions between the group of non-presbyopes and presbyopes. Results revealed that this was not the case as the difference was not statistically significant (*F*_(2, 215)_ = 1.844; *p =* 0.07). Furthermore, on average the trend was opposed to that expected as presbyopes tend to use smaller displays (mean: 13.8 cm, std: 1.8 cm) as compared to non-presbyopes (mean: 14.3, std: 1.7 cm).

We finally checked for any significant difference in reading distance and character size between presbyopes reporting no subjective difficulties in near vision (i.e., NAVQ score ≤ 10) and those reporting difficulties (i.e., NAVQ score > 10). We found that while reading distance was mostly unrelated to the score reported in NAVQ (*t*_(58)_ = -1.27; *p =* 0.209; d = -0.66) this was not the case for character size which was found to follow NAVQ scores (*t*_(58)_ = -2.31; *p =* 0.024) with participants with more difficulties in near vision adopting also larger character sizes (mean: 2.01 mm, std: 0.28 mm) as compared to participants with subthreshold scores (mean: 1.76 mm, std: 0.38 mm).

## Discussion

In the present study, we aimed at describing visual habits in the use of smartphones in a group of non-presbyopes and a group of presbyopes. Visual habits were defined as the average reading distance and character sizes used by the two groups while reading a simple text message on their smartphone.

Our results showed a significant difference in the usual reading distance adopted by the two groups as non-presbyopes were found to use closer working distances (mean = 33.4 cm; std = 7.6 cm) as compared to presbyopes (mean = 39.7 cm; std = 6.3 cm). Although this result was somehow expected because of a reduced lens accommodative power in presbyopes [5], it is interesting to note that the non-presbyopes used viewing distances that are shorter than those used during a classic optometric examination which are considered as a reference for reading printed materials (i.e., 40 cm). This result is not only in line with previous reports [3, 4] but also suggest the necessity to reconsider the habitual reading distance used in optometric examination. The reading distance adopted by presbyopes was instead very close to the value traditionally assumed for near work and mostly unrelated to the habitual correction (i.e., no correction, single vision lenses, progressive glasses and contact lenses).

Interestingly, we also found that presbyopes used, on average, a larger character size as compared to non-presbyopes. The two measurements (i.e., reading distance and character size) allowed us to infer the mean angular size used by the two groups expressed as the logarithm of the angle subtended by the smallest detail. This measurement revealed comparable reading acuities between the two groups which were however reached through different strategies. While non-presbyopes increase angular size by reducing reading distance, presbyopes increase character size, a behavior in line with previous reports [10] and likely due to the reduced lens accommodative power and to the near addition used. Furthermore, participants with suprathreshold scores in the NAVQ used significantly larger character sizes as compared to presbyopes reporting subthreshold scores. This highlights the relation between the use of larger characters size and subjectively experienced near vision difficulties. Importantly, we here described visual habits related to a specific behavior that is reading a text message on a smartphone. We predict that similar habits could be experienced also during other tasks employing the use of a smartphone, such as surfing on the web or looking up a contact, provided changes in character size are as well available for such tasks. However, we acknowledge that such prediction is not supported by current data and needs to be further tested.

It is worth to note that the present study lacks a measurement of the best near visual acuity to relate with angular size derived from viewing distance and character size. However, our sample was composed of participants with normal or corrected to normal vision and with no self-reported ocular pathologies. It is then reasonable to assume that, on average, the acuity of the sample was around 0 LogMAR (i.e., 0.4 M). We found that angular size was around 0.5 LogNAR (i.e., 1.3 M) in both non-presbyopes and presbyopes a value roughly three times larger from the expected best visual acuity. This result is in line with previous evidence comparing best visual acuity measurements with reading acuities, revealing that comfortable reading is achieved with a character size roughly three times larger [11, 12]. Importantly, the American National Standard Institute states that the minimum character height for text read at computer workstation should be between 22 and 30 minutes of arc. Here we showed that the habitual height for text read is on average smaller than the standard (mean: 17.06 min; range: 9.62 to 29.13 min) with a large percentage of participants (~77%) using heights smaller than 20 min. Therefore, the height of the text used is, in most of the cases, smaller than that recommended by the standards. This condition, together with the higher accommodative and vergence demand related to the lower reading distances adopted by non-presbyopes should be seriously considered during optometric examinations. Given the prolonged daily usage of smartphones, especially in younger individuals, we could expect an exacerbation of visual fatigue symptoms as compared with conditions of longer viewing distances used for printed materials. Although previous works showed that symptoms of asthenopia were not associated with abnormal accommodative responses [13], other works reported an increased lag of accommodation in subjects with high discomfort levels that manifested after extended viewing [14, 15]. A comprehensive assessment of accommodation and vergence system should then be included as a further visual examination for those users spending several hours a day on digital screen [16].

On the other hand, we found that presbyopes tend to increase the size of the characters to achieve good reading performances. Nevertheless, this behavior is not accompanied with the use of larger screen sizes as we here found no difference in adopted screen size in presbyopes and non-presbyopes. A comfortable reading requires not only the use of a correct character size but also a minimum number of character per line [17]. As a consequence, the use of small digital displays together with large character sizes can produce an uncomfortable reading which could significantly affect reading speed. For this reason, it would be recommendable that near vision difficulties are overcome not only by increasing the character size but also with the use of larger displays.

In conclusions, the present study showed that visual habits in the use of smartphones differ substantially across ages. We found that while non-presbyopes tend to use short working distance with small character sizes, presbyopes use higher working distances and a larger character size. We here lacked a comparison between smartphone-based measurements and measurements obtained in a classical optometric examination (i.e., distance and near visual acuity, reading acuity) which would have provided us with an evaluation of consistency between at distance and in person examinations. This limit was imposed by the Covid-19 pandemic period and we expect to be overcome by future studies aiming at fostering teleoptometry practices. Nevertheless, given the large-scale use of smartphones in our daily activities, we believe that the present results should be considered when determining the best near correction. For instance, determining the best reading acuity disregarding the reading distance adopted in daily activities could lead to an under- or over-compensation of the visual defect producing a relative discomfort during smartphone usage. Furthermore, the use of larger character size can represent a valid strategy to diminish the addition needed by presbyopes in near vision and, in turn, to reduce common issues reported by multifocal or progressive glasses users. All these points can be considered for future research leveraging on current data.
